# Ensemble of transformers for depression emotion classification

**DOI:** 10.1007/s11571-026-10444-0

**Published:** 2026-04-09

**Authors:** Furkan Kasap, Sevinç İlhan Omurca, Ekin Ekinci

**Affiliations:** 1 Computer Engineering Department Faculty of Engineering, Kocaeli University, Kocaeli, Turkey; 2 Computer Engineering Department Faculty of Technology, Sakarya University of Applied Sciences, Sakarya, Turkey

**Keywords:** Depression, AI in psychology, Large language models, Ensemble learning, Transformer-based ensemble, Encoder-only transformers, Multi-label classification

## Abstract

People act on their emotions even in the most rational decision-making mechanisms in their lives. Emotions are powerful motivators that profoundly influence human behavior and social interactions. Human emotions tend to co-occur. Analyzing emotions with this co-occurrence in mind may lead to more accurate insights for addressing various mental health issues. A good example of this is the analysis of depression, which often involves a complex interplay of multiple interrelated emotions rather than a single, isolated feeling. This paper provides a comprehensive analysis of co-occurring emotions in depression by using artificial intelligence methods. We have proposed a transformer-based ensemble model that predicts multiple emotional tendencies associated with depression based on the public DepressionEmo dataset of user posts associated with depression. DistilBERT, RoBERTa, Mental-BERT, Mental-RoBERTa, and DeBERTa are used as pre-trained transformers. The heterogeneous ensemble learning architecture developed using stacking and majority voting methods improves the individual prediction performance of the transformer architectures. Our study is the first to apply the transformer ensemble to the DepressionEmo dataset to identify multiple emotions in an individual’s textual psychological posts. Experimental results demonstrate that ensemble-based approaches provide more consistently improved performance compared to individual transformers, particularly in terms of macro-averaged F1 scores under conditions of class imbalance. Among ensemble learning approaches, the highest performance was achieved with Stacking-FFNN, which achieved 0.8121. These ensemble approaches consistently outperformed the strongest individual model, demonstrating the effectiveness of ensemble learning in improving depression emotion classification.

## Introduction

Depression is a prevalent mental health disorder that affects approximately 280 million people worldwide (World Health Organization, 2023). It is characterized by persistent sadness, a loss of interest in previously enjoyable activities, disturbances in appetite and sleep, reduced energy levels, and difficulty concentrating (Abbas et al. 2024). Depression may arise from prolonged experiences of emotional emptiness, sadness, stress, and anger, and is associated with an elevated risk of suicide (Rahman et al. 2024). Beyond its impact on the individual, depression often has adverse effects on family members and close interpersonal relationships. Therefore, this disease requires effective intervention and early diagnosis and treatment are very important for effective intervention.

Clinical assessments play a primary role in the diagnosis of depression, with traditional methods mainly relying on structured assessments and self-reported questionnaires (Kumar et al. 2024). However, traditional methods of diagnosing depression have several limitations. Contextual influences, the patient’s current psychological state, the quality of the clinician-patient relationship, emotional lability, previous clinical experiences, and memory biases can all significantly influence individuals’ responses (Phiri et al. 2025). Furthermore, individuals may be unaware of their depressive symptoms or embarrassed to disclose them, which may reduce their willingness to seek professional help. In fact, many individuals tend to avoid consulting healthcare professionals in the early stages of depression. In addition, face-to-face diagnostic approaches are often time- and cost-intensive. Therefore, there is a growing need for effective and scalable strategies that can facilitate early detection and diagnosis of depression.

In recent years, social media has emerged as a significant medium through which individuals express their emotions, thoughts, and personal experiences. The opportunity to communicate anonymously or under a pseudonym often reduces social inhibition, enabling users to share mental health-related content—such as symptoms of depression—more openly than they might in face-to-face interactions. Survey findings indicate that adolescents and young adults, in particular, predominantly use social media to express their emotions (Chiong et al. 2021). This dynamic presents a valuable opportunity for researchers and clinicians to detect early signs of depression by analyzing user-generated content on social media platforms. However, manually identifying whether an individual is experiencing depression, as well as determining the specific type of depression through social media platforms, remains a challenging task that demands significant human effort and resources.

Although machine learning (ML) has been successfully applied for many years in the classification of textual data, traditional approaches often rely on manual feature extraction (Thekkekara et al. 2024; Pande et al. 2024; Adarsh et al. 2023; Angskun et al. 2022). While these methods have demonstrated effectiveness in analyzing social media content for signs of depression, they exhibit notable limitations. In particular, their dependence on shallow linguistic features restricts their ability to capture the deeper and more nuanced expressions of depression. In contrast, Large Language Models (LLMs) are capable of automatically learning rich contextual representations and generating sentence or document-level embeddings, thereby eliminating the need for manual feature engineering. Moreover, LLMs have emerged as a transformative technology in text classification tasks, offering a powerful alternative to traditional ML methods by leveraging their ability to automatically capture complex semantic and contextual patterns (Dennstädt et al. 2024).

However individual LLM capabilities have been frequently evaluated in the literature; the inherent drawbacks of these models indicate that studying the behavior of LLM ensembles remains a critical and under-researched topic (Willis et al. 2025). In line with this research gap, it is important to recognize that while the recent success of LLMs has made them powerful tools for multifaceted feature extraction tasks, each model also exhibits limitations due to differences in architecture, diversity of training data, and the influence of hyper-parameters. These factors lead to challenges such as biases, inconsistent output, limited context management, and hallucinations when used in isolation, while simultaneously revealing their complementary potential (Ashiga et al. 2025). Therefore, developing a collaborative and integrated ensemble approach that preserves the strengths of individual LLMs while balancing their weaknesses is critical for achieving more reliable, diverse, and high-quality outputs (Fang et al. 2024a). However, LLMs typically produce solutions to natural language processing (NLP) tasks through text generation by giving a sequence of tokens as output (Fang et al. 2024b). This approach limits the direct applicability of classical ensemble methods developed for classification to LLMs. Therefore, the development of new ensemble strategies tailored to the characteristics of LLMs has become essential (Lu et al. 2024).

Ensemble learning strategies allow for the development of more successful classifiers by combining multiple learning models with different protocols. This learning principle is well-suited for tasks such as psychological assessments, where decision-making is highly subjective. In this study, we handle the 8-label multi-label text classification task, where each input instance is associated with one or more labels representing depression-related emotional states. Each label is treated as an independent binary classification problem, allowing the model to capture the co-occurrence of multiple emotional states within a single user post. To address this gap in the literature, the proposed study utilizes specialized transformer models such as Mental-BERT and Mental-RoBERTa, specifically tailored for mental health-related NLP tasks, in addition to general-purpose transformer models such as DistilBERT, RoBERTa and DeBERTa. Each base model that is a component of the ensemble learning model provides a distinct contextual representation of the input text, and their predictions are aggregated via soft voting, thereby enabling robust and generalized performance in identifying multiple co-occurring emotional states. Unlike soft or hard voting approaches that rely on fixed or uniform weighting schemes, the proposed stacking strategy enables a learnable and label-specific combination of model outputs. Each base model that is a component of the ensemble learning model provides a distinct contextual representation of the input text, and their predictions are aggregated via soft voting, thereby enabling robust and generalized performance in identifying multiple co-occurring emotional states. This is particularly advantageous in multi-label mental health classification, where different models may perform better for different emotional states. As a result, the stacking ensemble can more effectively exploit the complementary strengths of the base models. To the best of our knowledge, this study is the first to apply a supervised transformer-based stacking ensemble to the DepressionEmo dataset (Rahman et al. 2024) for multi-label emotion classification. To comprehensively assess the performance of the proposed multi-label ensemble framework, we evaluated it using several standard metrics, including accuracy, precision, recall, and F1-score. This study presents a detailed account of our methodology and results, aiming to advance the discourse on depression detection via social media and address persistent limitations.

Consequently, the main contributions of the paper are summarized as follows:

- We propose an artificial intelligence framework that predicts multiple emotions simultaneously from an individual’s text-based psychological posts.
- To the best of our knowledge, this study is the first to apply an ensemble of transformers on the DepressionEmo dataset, which was specifically prepared for multi-labeled emotion classification in the field of mental health.
- To contribute to the diversity of the ensemble model, specialized transformers (Mental-BERT and Mental-RoBERTa) that capture clinical nuances and domain-specific linguistic patterns, and general-purpose transformers (DistilBERT, RoBERTa and DeBERTa) have been combined as base classifiers. In our proposed framework, the stacking ensemble model combines emotion-label-specific class probabilities of five distinct transformer architectures.
- In a multi-emotion prediction task where emotions cannot be fully isolated, we demonstrated that the meta-learner in the stacking ensemble, which has the ability to learn the final output as a mixture of base transformer models, significantly outperforms the traditional majority voting and soft voting method, which combines all transformers by assuming that each transformer is “equally good” at every emotion.

## Related works

Detection of depression from texts such as social media posts, chat histories, clinical notes, Electronic Health Records (EHRs) or personal blogs is an important topic that attracts researchers’ attention and is intensively studied in the field of NLP (Palmon et al. 2021; Hamäläinen et al. 2021; Liu 2024; Liu et al. 2024; Vance et al. 2025; Yoo et al. 2025). However, the majority of studies in the literature are addressed within the framework of single-label classification, where only a single label is assigned to each example. These approaches usually classify expressions only as “depressed” or “not depressed/control” or classify them according to a single depression symptom (Tong et al. 2022; Cha et al. 2022; Beniwal and Saraswat 2024; Shah et al. 2025). However, depression is a multidimensional disorder as defined in clinical criteria such as DSM-5 and includes different symptom clusters. Therefore, more recent studies address depression with this multidimensional structure and tend to use multi-label classification methods with the assumption that expressions can carry more than one symptom at the same time.

Detection of depression from various sources such as Reddit, Twitter, suicide notes, hospital records and psychiatric social media content using multi-label classification methods has become one of the important research areas in recent years. In studies conducted on the DepressionEmo dataset consisting of Reddit posts, the BERT-uncased model demonstrated superior performance, achieving an overall F1-macro score of 78% and 85% for suicidal ideation detection (Rahman et al. 2024). On the same dataset, the contrastive learning method integrated with the self-augmentation approach and the RoBERTa model achieved an F1 score of 78.16% (Khan et al. 2024). KoBERT-based models developed based on DSM-5 diagnostic criteria have produced remarkable results in terms of clinical accuracy, reaching 97.47% and 100% F1-macro scores on both hospital data (Park et al. 2024) and Everytime platform data (Park et al. 2023), respectively.

In Twitter data, RankSVM and LSTM-attention based models were used on two different multi-emotion datasets with 9 and 16 labels, and F1-macro values ​​of 46.85% and 40.20% were obtained, respectively (Farruque et al. 2019). The CMSEKI multitask learning system developed on a historical dataset consisting of suicide notes performed depression, emotion and multiple emotion classification together with 74.35% accuracy (Ghosh et al. 2022). The Bi-LSTM-CNN model trained on social media texts with psychiatric content achieved 71% F1-macro score (Wu et al. 2020). In studies based on electronic health records, advanced models such as BioBERT, XLNet and Bio_ClinicalBERT achieved 81% F1 success (Zandbiglari et al. 2025).

In recent studies, LLMs are becoming increasingly common in multi-label classification tasks. For example, Llama-3 70b, GPT-4o-mini and Phi-3.5-MoE models have yielded successful results on Reddit-sourced data; the GPT-4o-mini model achieved 73% F1 score [8]. In a study conducted on dialogue-based data, LLMs were used only for feature extraction in a highly explainable multi-label classification system and achieved 90% accuracy (de Arriba-Pérez et al. 2024). Finally, the RoBERTa-based deep learning model developed on the MOSS-6 dataset obtained from Korean online mental health communities successfully classified six different types of social support seeking, achieving 72.4% F1 for 6 labels and 83.2% F1 for 3 labels ​​ (Kim et al. 2025). These studies reveal that transformer-based models and LLMs provide powerful and scalable solutions for multi-label sentiment and depression analysis in both social media and clinical texts. The related literature is concisely reviewed in Table 1.

**Table 1:** Comparative summary of datasets, methods, and results in multi-label depression classification literature

References	Dataset	Source	Number of Labels	Method	Results	Performance
(Rahman et al. 2024)	DepressionEmo	Reddit user posts	8	SVM, LightGBM, XgBoost, GAN-BERT, BERT-cased, BERT-uncased, BART, T5-small, T5-base	BERT-uncased gave the best results in terms of both success and parameter efficiency. DepressionEmo was particularly effective in recognizing the emotions of individuals showing symptoms of depression; the highest F1 Macro value was obtained in detecting suicidal intent.	Overall: 0.78 F1 Macro Suicidal Intent: 0.85 F1 Macro
(Khan et al. 2024)	DepressionEmo	Reddit user posts	8	Contrastive Learning+RoBERTa	In this study, a new approach based on self-augmentation is proposed, where Contrastive Learning is integrated into the language modeling process and the latent representations of the model are enriched with their own outputs.	78.16% Average F1 Score
(Violides et al. 2024)	DepressionEmo	Reddit user posts	8	Fine-tuned RoBERTa	In this study, authors used RoBERTa by fine-tuning it.	81.00% Average F1 Score
(Younas et al. 2025)	DepressionEmo GoEmotions Multi-label Dataset	Reddit user posts English Reddit comments Twitter	8 7 6	Stacked LSTM	In their study, the authors used the Stacked LSTM architecture. The model included encoder-decoder layers to capture short- and long-term dependencies between emotions, and a time-distributed dense layer to distinguish the specific features of each emotion.	Depression Emo: 0.70 Average F1 Score GoEmotions: 0.50 Average F1 Score Multi-label Dataset: 0.62 Average F1 Score
(Park et al. 2024)	Depression disorder representation data	Gangbuk Samsung Hospital	9	KoBERT Learning	In their study, the authors proposed a model that can detect depression in texts based on the DSM-5 diagnostic tool widely used in the medical field. They analyzed the correlation between nine DSM-5 depressive disorder diagnostic criteria and integrated these findings into their model, aiming to reduce the workload of physicians in the diagnosis of depressive disorders.	97.47% Average F1 Score
	Wellness conversation script dataset	Gangnam Severance				
	Everytime crawling data	Posts from Hankuk University of Foreign Studies				
(Farruque et al. 2019)	Cleaned Balanced Emotional Tweets	Twitter	9 and 16	RankSVM, LSTM-Att-WE	In their study, the authors used RankSVM and LSTM Attention methods in multi-label emotion mining tasks on two different multi-label datasets, one containing nine basic emotions and the other enriched with seven new emotions. In this process, they also used two widely used feature extraction methods namely bag-of-words and word embedding. Experimental results showed that LSTM Attention with word embedding methods gave the best results.	9 Emotion Dataset: 46.85% F1 Macro 16 Emotion Dataset: 40.20% F1 Macro
(Ghosh et al. 2022)	Suicide Notes Database	The dataset was collected between 1950 and 2011 by Dr. Edwin Shneidman and Cincinnati Children’s Hospital Medical Center and is derived from texts written by individuals before they committed suicide.	15	Cascaded Multitask System with External Knowledge Infusion (CMSEKI)	In their study, the authors proposed three different multi-task frameworks that tackle depression, emotion, and multi-label emotion recognition tasks together. These frameworks are based on the adaptation of a current approach to a more complex, stepwise version, the use of a network structure infused with external information, and strategies to improve performance in the multi-label emotion recognition task.	74.35% Accuracy
(Wu et al. 2020)	Psychiatric Social Texts	PsychPark website	7	Bi-Directional LSTM-CNN	In the study, a classification model was built using word embeddings, CNN and LSTM based structures in order to detect multiple emotional labels on social media texts with psychiatric content.	0.71 F1 Macro
(Zandbiglari et al. 2025)	MIMIC-IV critical care database	The dataset was obtained from de-identified health data of more than 40,000 critical care patients treated at Beth Israel Deaconess Medical Center between 2008 and 2019.	6	BERT, RoBERTa, XLNet, BioBERT, Bio_ClinicalBERT	In their study, the authors developed a robust NLP framework that uses detailed labeling processes and transformer-based language models to detect suicidal behaviors in Electronic Health Records (EHR). They also proposed a semi-automatic NLP pipeline that can extract sentences from unstructured EHR texts, generating a high-quality dataset and enabling more detailed analysis of suicidal behaviors.	0.81 Average F1 Score
(Hassan et al. 2024)	Depseverity-Dreaddit	Reddit user posts	4	Llama-3 70b, GPT-4o-mini, Phi-3.5-MoE	In their study, the authors utilized LLMs in the transition process from single-label classification to multi-label classification; by trying various routing techniques, they demonstrated that LLMs exhibited consistent and high performance despite the increase in the number of labels and classes.	Llama-3 70b: 0.71 Average F1 Score
	Reddit Mental Health Dataset		6			GPT-4o-mini: 0.73 Average F1 Score
(Park et al. 2023)	Everytime crawling data	Posts from Hankuk University of Foreign Studies	9	KoBERT Learning	The authors used data obtained from the Everytime platform to analyze the psychological states of young individuals. They increased the clinical applicability of the method by developing a prediction method compatible with the DSM-5 depressive disorder diagnostic criteria. They also evaluated the statements according to the nine DSM-5 criteria with the multiple labeling method and calculated the probability of corresponding to each criterion.	1.00 F1 Macro
(de Arriba-Pérez et al. 2024)	Dialogues + clinical data	Full dialogues between users and the Celia chatbot	14	Multi-label ML + LLMs	The authors proposed an ML system with high explainability and increased accuracy using LLMs for feature extraction only for multi-label classification of anxiety and depression. The study provides an important contribution towards the reliable and scalable use of Artificial Intelligence-based solutions in the healthcare field.	90.00% Accuracy
(Kim et al. 2025)	MOSS-6	Mental Health and Depression Board within EveryTime online community	6	RoBERTa embeddings + CNN + BiLSTM	The authors presented a new Korean language multi-label dataset, MOSS-6, that included six different types of support to analyze individuals’ social support seeking in online mental health communities. This study was one of the first to demonstrate the predictability of these behaviors across all types of social support.	6 label dataset: 0.724 Average F1 Score 3 label dataset: 0.832 Average F1 Score

Compared to previous studies, our work addresses a significant gap in the literature on multi-label depression emotion classification by moving beyond single-model and task-specific architectures to offer a unified ensemble-based learning framework. Existing studies primarily focus on individual deep learning approaches, such as transforming models or recurrent neural networks, for multi-label emotion detection. However, they often treat the outputs of these models independently and underutilize the complementary potential of different representational strengths. In contrast, this study systematically combines general-purpose transforming models tailored to the mental health domain with a stacking-based ensemble learning strategy. This approach enables more effective modeling of inter-label relationships, reduces model-specific biases, and improves overall generalization performance. The consistent performance improvements achieved compared to robust multi-label baseline models on the DepressionEmo dataset demonstrate that the proposed approach offers a scalable and methodologically sound solution for multi-label depression analysis.

## Materials and methods

### Dataset

The public DepressionEmo dataset which was created by Rahman et al. (2024) is used for the experiments. The dataset sourced from Reddit, includes the user posts associated with depression and is partitioned into a training set comprising 5,131 instances and a test set consisting of 906 instances. Examples of user posts and their associated emotion annotations are listed in Table 2. Each textual user post in the dataset was labeled with one or more of the eight depression-related emotions such as anger, cognitive dysfunction, emptiness, hopelessness, loneliness, sadness, suicide intent and worthlessness. The emotional distribution across texts are as follows, sadness comprising the largest share at 21.2%, followed by hopelessness at 19.1%, worthlessness at 13.6%, loneliness at 12.7%, anger at 11.1%, emptiness at 10.3%, suicide intent at 6.8%, and finally with the smallest portion, cognitive dysfunction at 5.2%. Given the class distributions, DepressionEmo stands out as an imbalanced dataset for the classification task.

**Table 2:** Sample user posts with emotion annotations

Text (Title and Post)	Date	Emotions
Found out something awful ### My mum had a boyfriend when I was around 6 or 7. She met him while she was volunteering at a prison. He was incarcerated for 15 years following a spate of armed robberies. When he was released she promptly moved him in with us. I found out that the only reason he was released is because she told the parole board that he could move in with us. What kind of a parent does that? A few years later after we escaped from him, he ended up shooting a guy 4 time’s in the kneecaps in a McDonald’s parking lot and was sentenced to another decade or so in prison. Guns aren’t easy to come by in Australia.	2020-06-28 11:16:59	anger, hopelessness, sadness
Done ### I’m writing this as I sit on the side of the road under a bridge of a busy highway. I can’t carry on like this anymore. I’ve tried so hard for so long to not let things bother me but I just can’t anymore. It’s just one thing after another I cant take another thing. People will say that she was always so helpful and never let anyone down, she would give you the shirt off of her back. They will say I don’t know why she didn’t reach out. I’ll tell you why because people don’t actually care. They are nosey. I know I don’t fit in. I know the people in the “friends” group I have aren’t really my friends. I know they all talk and laugh about me behind my back. As I sit here, there is not a single person I can talk to, reach out to or ask for help from. The only reason people will miss me is because I’m not there when they need something.	2022-08-26 08:53:35	loneliness, hopelessness, sadness, worthlessness, anger, emptiness
wtf am I never not tired? ### no matter where I’m at or what I’m doing, I’m never not tired. No amount of doing things or not doing things matters. Wake up tired, eat breakfast tired, go to work tired, watch tv tired. I fucking hate this. I hate everything about this. How is anyone supposed to make progress when it’s an endless cycle? The only thing that doesn’t change is depression and misery.	2023-10-10 18:50:23	hopelessness, worthlessness, sadness, anger

**Fig. 1:**
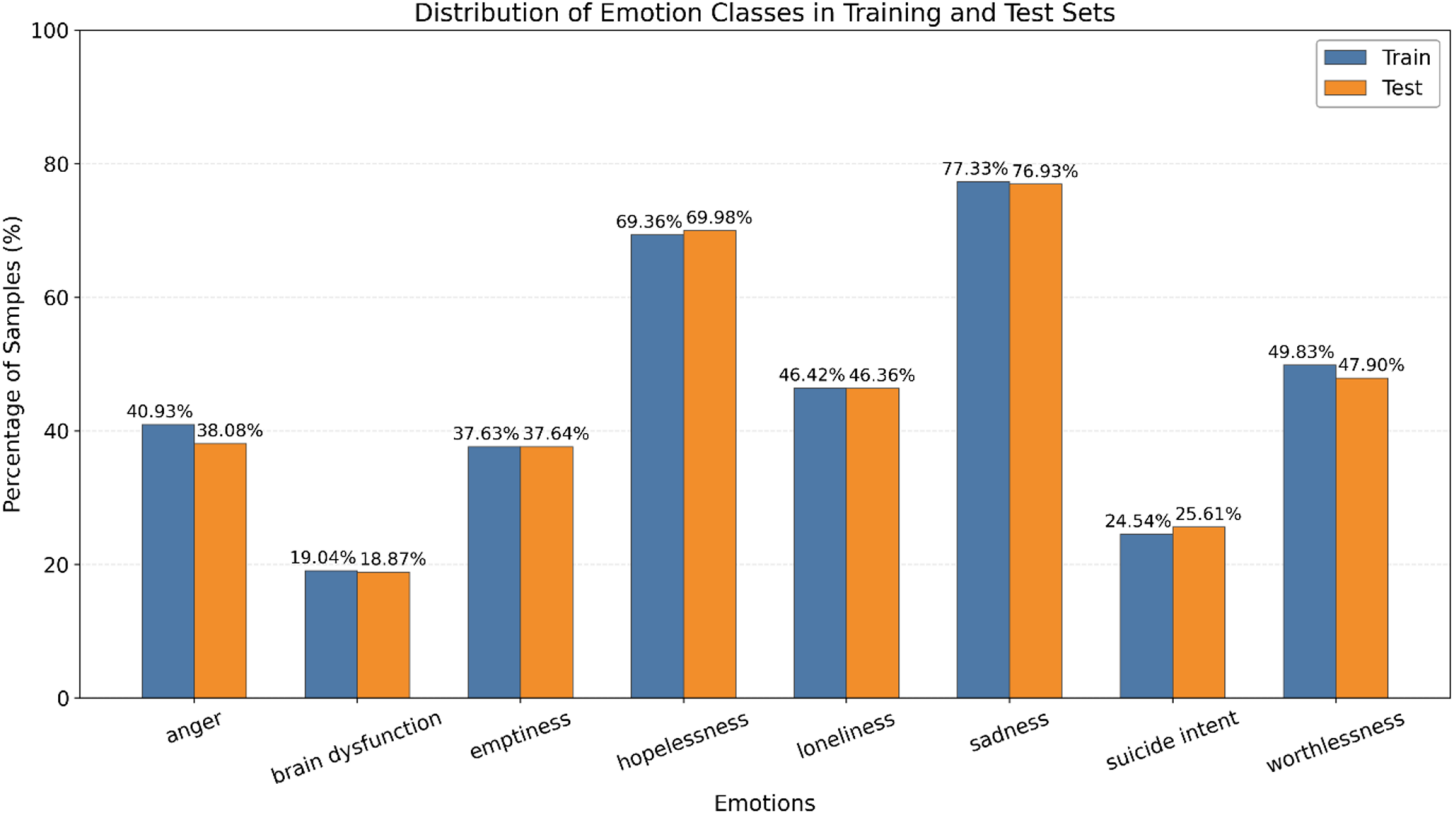
Comparison of class distributions between the training and test sets

Given these distributions, DepressionEmo is clearly an imbalanced dataset for the classification task. Beyond class imbalance, the multi-label nature of the dataset presents another important characteristic worth examining. The distribution of labels per sample was analyzed in both the training and test sets. In the training set, the largest proportion of samples contained a single label (18.6%), followed by samples with two (15.3%), three (15.4%), four (15.7%), and five (15.1%) labels. The share of samples decreased considerably for those with six (10.6%), seven (6.3%), and eight (2.9%) labels. A similar trend was observed in the test set: single-label samples accounted for 20.0% of the data, while samples with two, three, four, and five labels represented 15.0%, 13.9%, 17.4%, and 13.9%, respectively. The proportions declined further for six (11.0%), seven (5.6%), and eight (3.1%) labels. These findings indicate that although the dataset supports multi-label assignments, the majority of samples contain between one and five labels, with a sharp drop in frequency as the number of labels increases. Comparison of class distributions between the training and test sets is presented through Fig. 1. These distributions represent sample-based prevalence rates, showing how many different samples each label appeared in.

### Large language models

In this paper, experiments were conducted using different language models, such as DistilBERT, RoBERTa, Mental-BERT, Mental-RoBERTa-base and DeBERTa, to provide a comprehensive evaluation. The respective language models were chosen on the basis of pre-trained transformers in clinical (or similar) domains that have produced effective results in depression diagnosis in the literature to facilitate robust comparison and generalizability of the findings.

Ensemble learning strategies allow for the development of more successful classifiers by combining multiple learning models with different protocols. This learning principle is well-suited for tasks such as psychological assessments, where decision-making is highly subjective. Based on this fundamental motivation, the proposed study utilizes specialized transformer models such as Mental-BERT and Mental-RoBERTa, specifically tailored for mental health-related NLP tasks, in addition to general-purpose transformer models such as DistilBERT, RoBERTa and DeBERTa.

In this study, we combined general and specialized transformers and selected MentalBERT and MentalRoBERTa, specialized transformers trained to better capture linguistic and psychological patterns commonly used in the mental health field. One reason for choosing these models is that they are specialized transformers trained to better capture linguistic and psychological patterns commonly used in the mental health field. Another reason is that the training corpus used to adapt these models to the mental health domain was sourced from social media platforms, where individuals are more inclined to express their emotions openly and where such content is more accessible.

#### DistilBERT: a distilled version of BERT

DistilBERT (a distilled version of BERT) is a lightweight BERT-based model developed to reduce the model size and computational cost of BERT models while retaining their performance (Sanh et al. 2019). DistilBERT achieves this by using knowledge distillation technique where a smaller model learns from a large and pre-trained language BERT model. The key objectives of DistilBERT are to achieve computational efficiency, attain faster inference speeds, and retain the performance comparable to BERT.

#### RoBERTa (robustly optimized BERT approach)

RoBERTa (Robustly Optimized BERT Approach), trained version of BERT with an improved recipe developed by Facebook AI, designed to enhance language model performance through more effective training strategies. Compared to BERT training, RoBERTa is trained for more epochs using more training data and with larger batch sizes. The next sentence prediction objective of BERT is removed. Utilizing longer text sequences in training. Dynamically changing the training data’s masking pattern (Liu et al. 2019).

#### Mental-BERT and mental-RoBERTa

MentalBERT and MentalRoBERTa are two domain adapted language models developed for mental health NLP tasks and pre-trained on a corpus of user posts collected from the Reddit social media platform focused on mental health discussions (Ji et al. 2021). While MentalBERT shares the same features as BERT in terms of its architectural structure and pre-training tasks, MentalRoBERTa shares the same features as RoBERTa in terms of its architectural structure and pre-training tasks (Neagu and Spinu 2024). This domain-adaptive pre-training process ensures that models effectively capture domain-specific knowledge, emotional indicators, and language patterns associated with depressive symptoms and psychosocial distress related to mental health conditions (Pradnyana et al. 2025).

#### DeBERTa (decoding-enhanced BERT with disentangled attention)

DeBERTa (Decoding-enhanced BERT with disentangled attention) is a Transformer-based neural network language model and aims to improve BERT and RoBERTa models with two innovative techniques (He et al. 2020). In the first technique, the disentangled attention mechanism, each word is expressed with two separate vectors representing both its content and position. Attention weights between words are calculated with matrices created separately for content and relative position information. The second technique is the enhanced mask decoder structure. A key feature of the model, the disaggregated attention mechanism, focuses attention on relative positions; however, the model also requires absolute position information. To achieve this, DeBERTa incorporates absolute positional embeddings during the decoding phase, immediately before the model predicts masked tokens during pre-training. This allows for the learning of more robust contextual representations.

**Enhanced Mask Decoder (EMD)** The second innovation, the Enhanced Mask Decoder, addresses the limitations of relative positions in understanding global sequence structure. During the pre-training stage, DeBERTa incorporates **absolute position embeddings** right before the softmax layer for predicting masked tokens. By providing the model with the absolute location of tokens at the final decoding stage, the EMD ensures that the representation learning process compensates for the relative-only information used in the earlier layers. These two techniques collectively enable DeBERTa to achieve superior efficiency and accuracy in downstream discriminative tasks compared to its predecessors.

### Ensemble learning

In analyzing a mental health condition such as depression, which has multiple causes and affects people’s emotional states in multiple ways, it is very important to take into account the tendency for multiple emotions to occur together. With this basic motivation, we have investigated the use of LLMs as stand-alone models for mood prediction of depression, as well as ensemble learning models that combine these models. With this motivation, we explored the effectiveness of majority voting (Lam and Suen 1994) and stacking (stacked generalization) (Wolpert 1992) methods in depression emotion prediction.

Majority voting is the simplest way to combine several base learners in ensemble learning. Combining diverse base learners, ensemble hypotheses can achieve an accurate prediction result. The voting mechanism, a mechanism for aggregating multiple diverse classifiers, serves as a central mechanism in ensemble classifiers. Hard voting and soft voting methods are two primary majority voting strategies. In hard voting, the ensemble classifier combines the classification decisions of base models for a given test input, not the probabilities. The resulting class with the most votes is determined as the final ensemble decision. Soft voting, on the other hand, combines the confidence levels of each diverse base classifier. Rather than relying on the classification decisions of base learners, soft voting takes into account probability distributions each model outputs for all possible classes. The class label with the highest aggregated probability is the final ensemble decision.

We assume that, the class labels are obtained from the base classifiers, and the decision label of the $\:k$-th classifier is $\:{l}_{kj}\in\:\left\{\mathrm{0,1}\right\},k=1,...,K$ and $\:j=1,...,C$; where $\:K$ is the number of base classifiers and $\:C$ is the number of class labels. Suppose the $\:k$-th base classifier predicts the desired class label $\:{d}_{j=J}$ then $\:{l}_{kj}=1$ and 0, otherwise. The ensemble decision for each emotion label $\:\stackrel{\prime }{{y}_{j}}$ using majority voting (hard voting) is defined as in Eq. 1.

1 $$\:{\stackrel{\prime }{y}}_{j}=\left\{\begin{array}{c}1,\left({\sum\:}_{k=1}^{K}{l}_{kj}\right)\ge\:\frac{K}{2}\\\:0,\mathrm{o}\mathrm{t}\mathrm{h}\mathrm{e}\mathrm{r}\mathrm{w}\mathrm{i}\mathrm{s}\mathrm{e}\end{array}\right.$$

For the above explained classification representation, the kth classifier returns a vector of probabilities for an input data sample $\:x$, $\:\left[{p}_{k1},{p}_{k2},...,{p}_{kC}\right]$, where the $\:{p}_{kj}$ is the output probability of $\:x$ belonging to class $\:j$. The final ensemble probability score is calculated as in Eq. 2.

2 $$\:{S}_{j}=\frac{1}{K}{\sum\:}_{k=1}^{K}{w}_{k}{p}_{kj};\:j=\left(\mathrm{1,2}, \ldots ,C\right)$$

here, $\:{w}_{k}$ defines the weight assigned to the $\:k$-th base classifier.

The final ensemble hypothesis for soft voting is determined by selecting the label with the highest value among these C average probabilities. The final ensemble hypothesis for soft voting is given in Eq. 3.

3 $$\:{\stackrel{\prime }{H}}_{softVoting}={argmax}_{j}maxSj ; (j=1, \ldots ,C)$$

Stacking offers a more efficient way to reduce the deviation of the generalizer from the provided learning set (Haque et al. 2025; Siddiqui et al. 2025). To ensure this, first ensemble of classifiers is built, then outputs of the ensemble are used as inputs to a second level classifier to learn the decision boundary between the ensemble outputs and the actual class label (Polikar 2006). The final prediction hypothesis for stacking is defined as follows through a matching function $\:{f}_{s}$.

4 $$\:\stackrel{\prime }{l}={f}_{meta}\left(Z\right)$$

here, Z is the concatenated vector of probability scores generated by 5 base classifiers for 8 labels which is represented as in Eq. 7.

5 $$\:Z=\left[{p}_{11},{p}_{12}, \ldots ,{p}_{18},{p}_{21}, \ldots ,{p}_{28},{p}_{31}, \ldots ,{p}_{38},{p}_{41}, \ldots ,{p}_{48},{p}_{51}, \ldots ,{p}_{58}\right] \in {R}^{40}$$

## Proposed model

The main motivation of our proposed model is to effectively integrate ensemble learning strategies with transformer-based encoders to enhance the performance of multi-label emotion detection in depression-related texts. The proposed model addresses the inherent complexity of identifying a series of emotions present simultaneously within a single text that expresses a person’s emotional state. This is a task that traditional models often struggle with. In this section we describe the overall architecture of the proposed model. Then the model training of base LLMs is briefly explained. And finally, the meta feature extraction and stacked ensemble learning stage is explained.

### Architecture overview

While Autoregressive LLMs are trained to predict the next token given the previous context, enabling sequential generation, Autoencoding LLMs, in contrast, learn to produce bidirectional, context-aware embeddings of input sequences by reconstructing masked tokens.

While Holtzman et al. (2021) demonstrated that the -highest probability answer of an Autoregressive LLM may not be the right decision, our stacking motivation primarily relies on ensemble learning principles for multi-label classification. Our model, assuming that in calculating the probabilities of different emotions in an emotion-containing text, the emotion with the highest probability alone cannot reflect the valid emotion, and the probability values assigned to other emotions are equally important. Although this insight is framed in the context of autoregressive models, it is based on the premise that it can be similarly applied to autoencoding models such as BERT in classification or masked token prediction tasks, where the softmax output reflects the distribution over plausible alternatives. Holtzman et al. (2021) is therefore referenced only as a high-level intuition regarding the informativeness of non-maximal probabilities.

For a given text, we combine the event distributions of eight possible emotion classes in $\:N$ different LLMs, to generate the ensemble output vector $\:\stackrel{\prime }{y}$. Let $\:x$ denote a text from the emotion dataset $\:D$, which serves as input to the language model $\:L$, and let $\:\stackrel{\prime }{y}$ represent the corresponding target emotion vector (ensemble output vector). For the $\:i$-th transformer, the probability distribution is defined as $\:{L}_{ij}\left(x\right)={q}_{i}$, where $\:j=1,...,8$, and is used to generate $\:\stackrel{\prime }{y}$. We then define a pool of $\:N$ LLMs, denoted as $\:{L}_{1},...,{L}_{N}$, where $\:N=5$ in our experiments. To combine the probability distributions from the five LLMs, their emotion probability estimates are concatenated to form a new input vector for the ensemble model. Consequently, for five LLMs each predicting eight emotion classes, this results in a 40-dimensional input vector for the ML model. The final stacked hypothesis is defined by a matching function as in Eq. 6 aggregates the base LLMs’ outputs.

6 $$\:\stackrel{\prime }{y}={f}_{w}\left({q}_{1},...,{q}_{N}\right)$$

where, $\:{f}_{w}\left(.\right)$ denotes the Feed Forward Neural Network (FFNN) parameterized by $\:w$ as a stacked ensemble learner. In our study, we use a FFNN containing multiple layers of fully connected weights with ReLU activation functions. The final layer generates eight logit values, each corresponding to an emotion label. The model was trained using BCEWithLogitsLoss for the multi-label classification problem. In the inference phase, probability values are obtained on a label basis by applying a sigmoid function to each logit, and final predictions are generated by filtering these probabilities according to a predetermined threshold value.

**Fig. 2:**
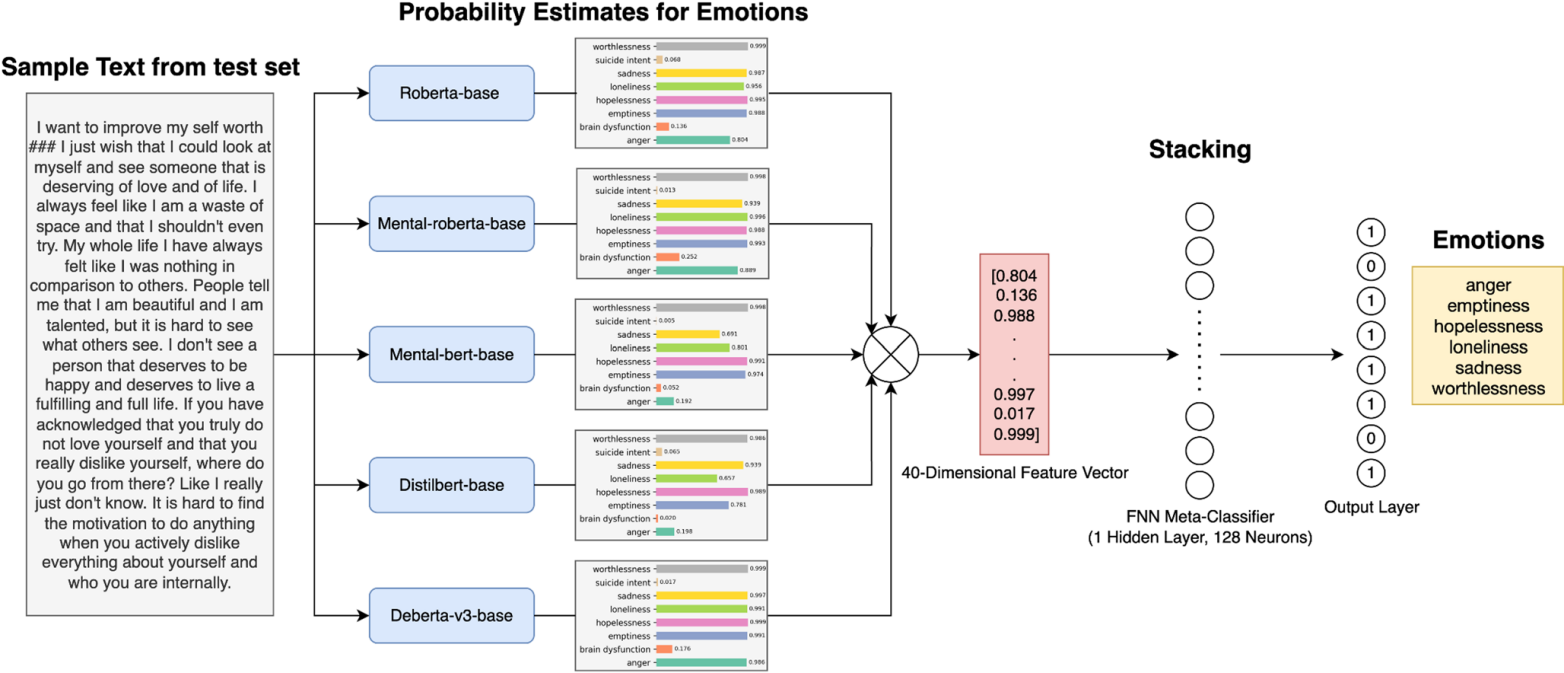
The architecture of proposed model

The proposed architecture is proposed in Fig. 2. The solution pipeline consists of two main stages. (1) training of 5 LLMs as base learners. (2) Training of the FFNN as the meta classifier of the stacked ensemble model.

In the first stage, each base model generates independent probability estimates for emotion labels of a given text. In the second stage, these probabilities are combined via a trainable stacking (meta-learner) approach, effectively leveraging the strengths and complementary predictions of different LLMs. This hybrid approach achieves higher and more reliable prediction performance than the performance of each model individually.

In other words, each LLM functions like an independent psychologist, providing its own assessment of the emotional state. The proposed meta-classifier then plays the role of a main evaluator, synthesizing these individual perspectives into a final decision. By integrating diverse viewpoints, the ensemble produces a more balanced and accurate judgment of the underlying emotions.

In ML, when constructing ensemble learning models and implementing a stacked ensemble model, the FFNN is a widely applied model in the base learner combining phase due to its ability to learn non-linear relationships between the outputs of different base learners. The reason for choosing FFNN as the meta-learner in our study can be explained as follows: the FFNN model allows for the effective capture of complementary patterns learned by individual transformer models. Especially in complex and subjective tasks such as psychological text classification, it is critical to effectively learn base classifiers using ML processes like FFNN, rather than combining them with simple strategies like majority voting.

### Base model development

The proposed ensemble model employs Mental-BERT, RoBERTa-base, DistilBERT-base, Mental-RoBERTa, and DeBERTa-v3-base as its base models. Each model was trained on different data subsets using a 5-fold cross-validation strategy, yielding five distinct instances per model. For the inference stage, the model instance (fold) that achieved the highest F1-macro score during 5-fold cross-validation was selected as the final model for each architecture. During training, a layer-wise dropout strategy was employed to enhance the models’ generalization ability and mitigate overfitting. In this approach, dropout rates are progressively increased from the shallow to the deeper layers, enabling more effective regularization, particularly in the deeper layers with higher expressive capacity.

The proposed ensemble model employs Mental-BERT, RoBERTa-base, DistilBERT-base, Mental-RoBERTa and DeBERTa-v3-base as base learners. Each base model is trained using 5-fold cross-validation. For the inference pipeline, the model instance (fold) that achieved the highest F1-macro score during 5-fold cross-validation was selected as the final model for each architecture. In other words, for each model, the “best fold” is selected solely based on the F1-macro score on the corresponding fold’s validation split. During the training of each base model, we apply a layer-wise dropout strategy to improve generalization and mitigate overfitting.

### Extraction of meta-features

To prevent data leakage, the meta-classifier was trained using an Out-of-Fold (OOF) prediction strategy. Each training sample was represented by a comprehensive 40-dimensional meta-feature vector composed of predictions from five distinct transformer models (8 emotion predictions × 5 models). These meta-features serve as informative inputs for capturing subtle differences and interactions among the models.

### Meta-learner training

An FFNN was selected as the meta-learner due to its ability to flexibly capture complex, non-linear relationships. The meta-learner is trained exclusively on the OOF meta-features and the corresponding training labels; no test samples are used during meta-learner training or model selection. The FFNN architecture consists of a single hidden layer with 128 neurons, followed by batch normalization and a ReLU activation function. The final layer outputs 8 logits (one per emotion label) and the network is optimized using Binary Cross-Entropy with Logits (BCEWithLogitsLoss). At inference, a sigmoid function is applied to each logit to obtain per-label probabilities, enabling multi-label classification. In our experiments, the FFNN-based stacking approach yielded stronger performance than logistic regression and gradient-boosted trees (XGBoost) as alternative meta-learners.

### Inference procedure

During inference, each test set samples are tokenized and passed through each base architecture to obtain per-label probability estimates. Meta-features for test samples are generated using the single best-performing fold model per base architecture (selected based on validation F1-macro within cross-validation), rather than averaging across all K folds. The resulting 40-dimensional vector is then fed to the trained FFNN meta-learner to produce final emotion predictions. Finally, a decision threshold of 0.45 is selected based on validation-set experiments as shown in Table 3.

**Table 3:** Threshold selection results for the stacking-FNN ensemble on the validation set

Threshold	F1 Macro	F1 Micro
0.30	0.8086	0.8430
0.35	0.8129	0.8479
0.40	0.8128	0.8496
**0.45**	**0.8103**	**0.8500**
0.50	0.8097	0.8509
0.55	0.8011	0.8468
0.60	0.7966	0.8450
0.65	0.7890	0.8395
0.70	0.7749	0.8290

According to Table 3, F1-macro reaches its highest value at the 0.35 threshold, while F1-micro reaches its highest value at approximately 0.50. However, in this study, threshold selection was based on a multi-objective evaluation aiming to achieve balanced performance between F1-macro and F1-micro, rather than targeting the maximum of a single metric. In this context, the 0.45 threshold was determined as the optimal equilibrium point that almost maximizes the F1-micro value while not causing a significant decrease in F1-macro. Due to losses in F1-micro at lower thresholds and decreases in F1-macro at higher thresholds, the 0.45 threshold offers a stable and balanced solution.

In summary, the hybrid model combines baseline model training enhanced with stepwise dropout, OOF meta-feature extraction, and an FFNN-based stacking mechanism. This approach significantly improves multi-label emotion detection performance on depression-related texts.

## Experimental study

### Experimental setup

In this study, five different pre-trained deep learning models obtained from the Hugging Face Transformers library were fine-tuned for the task of multi-label emotion classification. The models used include RoBERTa-base (Facebook 2025), which was pre-trained on large-scale English texts; mental-RoBERTa-base (Mental 2025a) and mental-bert-base-uncased (Mental 2025b), which were pre-trained on mental health forums and medical texts such as PubMed and PMC; distilbert-base (DistilBERT 2025), which is a distilled version of the BERT architecture, and deberta-v3-base (Microsoft 2025), developed by Microsoft, which offers improved performance in terms of context representation. RoBERTa has a 12-layer architecture with 768 hidden units and 12 attention heads, containing a total of 125 million parameters (HuggingFace, 2019). Both mental-RoBERTa-base and mental-bert-base-uncased include 12 layers and 768 hidden units; mental-bert-base-uncased has approximately 110 M parameters, whereas mental-RoBERTa-base has about 125 M parameters (Ren et al. 2024; Yang et al. 2025). DistilBERT-base consists of 6 layers, 768 hidden units, 12 attention heads, and approximately 66 million parameters (Hugging Face 2024). deberta-v3-base model is a 12-layer structure containing 768 hidden dimensions (Hugging Face 2022). It has a vocabulary of 128,000 words and contains 86 million parameters in the backbone section, while this structure allows it to reach 98 million parameters in the embedded layer. Each of these models was reconstructed with an eight-neuron output layer with a sigmoid activation function to predict eight different emotion labels (anger, cognitive dysfunction, emptiness, hopelessness, loneliness, sadness, suicide intent, worthlessness).

The hyper-parameters used during model training are as follows: mini batch size 8, maximum input length 256 tokens, learning rate 2 × 10^− 5^ and total epoch number 25. However, due to the early stopping, training usually ended between 6 and 10 epochs. The early stopping criterion was triggered when the validation loss did not improve for three consecutive epochs. We applied a layer-wise, progressive dropout schedule across the transformer encoder layers, with the dropout rate increasing linearly from 0.1 (shallow layers) to 0.3 (deeper layers) during fine-tuning. AdamW algorithm was used for the optimization process and the L2 weight decay value was left as 0.01 by default. The learning rate was set with the get_linear_schedule_with_warmup method along with a 10% warm-up process at the beginning of training. Gradient clipping was applied to prevent excessive gradient values ​​from negatively affecting the model performance and the maximum norm value was limited to 1.0. For reproducibility, we fixed the random seed to 42 for data splitting and model training.

In order to increase the reliability of the model performance, we performed 5-fold cross-validation using the MultilabelStratifiedKFold method, which performs data splitting by preserving the class distribution in multi-label tasks. This ensured a balanced distribution of labels in each fold, and the model weights from the epoch that achieved the highest validation F1 Macro score in each fold were saved. The final evaluations were performed using these best weights.

### Evaluation metrics

The experimental results are evaluated based on F1 Macro, Precision Macro, Recall Macro, F1 Micro, Precision Micro, and Recall Micro metrics. F1 Micro measures the overall performance of the model without considering class distinctions, whereas F1 Macro calculates the average of the F1 scores for each emotion class, offering a more balanced, class-specific evaluation. The equations for the F1 Micro and F1 Macro formulations calculated based on the precision and recall metrics are presented below.

7 $$\:{Precision}_{micro}=\frac{{\sum\:}_{c=1}^{C}T{P}_{c}}{{\sum\:}_{c=1}^{C}T{P}_{c}+F{P}_{c}}$$

8 $$\:{Precision}_{macro}=\frac{1}{C}{\sum\:}_{c=1}^{C}\frac{T{P}_{c}}{T{P}_{c}+F{P}_{c}}$$

9 $$\:{Recall}_{micro}=\frac{{\sum\:}_{c=1}^{C}T{P}_{c}}{{\sum\:}_{c=1}^{C}T{P}_{c}+F{N}_{c}}$$

10 $$\:{Recall}_{macro}=\frac{1}{C}{\sum\:}_{c=1}^{C}\frac{T{P}_{c}}{T{P}_{c}+F{N}_{c}}$$

In multi-label classification, $\:C$ denotes the total number of labels and N represents the number of instances in the dataset. For each label $\:c\in\:\{\mathrm{1,2},...,C\}$, the classification performance can be represented using standard confusion matrix components. $\:T{P}_{c}$​ denotes the number of true positives for label $\:c$, i.e., instances correctly predicted as belonging to label $\:c$. Similarly, $\:F{P}_{c}$​ refers to the number of false positives, representing instances incorrectly predicted as belonging to label $\:c$, while $\:F{N}_{c}$ denotes the number of false negatives, corresponding to instances that actually belong to label $\:c$ but were not predicted as such.

11 $$\:{F1}_{micro}=\frac{2\times\:Precisio{n}_{micro}\times\:Recal{l}_{micro}}{Precisio{n}_{micro}+Recal{l}_{micro}}$$

12 $$\:{F1}_{macro}=\frac{1}{C}{\sum\:}_{c=1}^{C}\frac{2\times\:Precisio{n}_{c}\times\:Recal{l}_{c}}{Precisio{n}_{c}+Recal{l}_{c}}$$

F1 Micro and F1 Macro performance metrics are the most suitable evaluation metrics for multi-label classification, as presented in Eqs. 11 and 12 (Hinojosa Lee et al. 2024). F1 Macro assigns equal importance to each class by computing the F1 score separately for each class and then averaging the results. The F1 Macro score provides an advantage in cases of class imbalance by ensuring that each class has an equal impact on the final metric. In contrast, F1 Micro aggregates the true positives (TP), false positives (FP), and false negatives (FN) across all classes to obtain a unified score. This method is beneficial when the focus is on overall system performance, particularly emphasizing the majority class while still accounting for the minority classes.

To provide a more comprehensive evaluation of the experimental results, in addition to standard Macro-F1 and Micro-F1 metrics, Jaccard index (Jaccard accuracy) was also used as an evaluation metric. Each textual item in the DepressionEmo dataset can be labeled with a set of eight distinct emotions. The Jaccard index evaluates the partial prediction performance of the model by measuring the intersection between the predicted label set and the true label set for each textual example. The formulation for calculating Jaccard Accuracy is defined in Eq. 13.

13 $$\:JaccardAccuracy=\frac{1}{T}{\sum\:}_{t\in\:T}\frac{{G}_{t}\cap\:{P}_{t}}{{G}_{t}\cup\:{P}_{t}}$$

here, |T| represents the number of samples in the dataset, G_t_ represents the true label set, and P_t_ represents the predicted label set.

A high Jaccard score indicates a higher overlap of predicted labels with the true labels, allowing for effective evaluation of model performance on multi-label datasets like DepressionEmo.

### Experimental Results

Table 4 summarizes the performance of the algorithms on multi-label depression emotion classification of user posts in terms of F1 Macro, Precision Macro, Recall Macro, F1 Micro, Precision Micro and Recall Micro.

**Table 4:** Performance comparison of text classification methods on the test set

Method	F1 macro	Precision macro	Recall macro	F1 micro	Precision micro	Recall micro	Jaccard accuracy
RoBERTa	0.7968	0.7776	0.8220	0.8298	0.8005	0.8613	0.6924
Mental-RoBERTa	0.7919	0.7579	0.8307	0.8213	0.7866	0.8592	0.6811
Mental-BERT	0.7836	0.7748	0.7952	0.8156	0.8033	0.8283	0.6740
DistilBERT	0.7629	0.7459	0.8011	0.8059	0.7609	0.8564	0.6604
DeBERTa-v3	0.7895	0.7549	0.8297	0.8230	0.7839	0.8662	0.6841
Hard Voting	0.8062	0.7852	0.8315	0.8377	0.8081	0.8696	0.7076
Soft Voting	0.8040	0.7847	0.8286	0.8370	0.8073	0.8690	0.7063
Stacking-LR	0.8027	0.7817	0.8313	0.8256	0.8103	0.8415	0.6833
Stacking-XGB	0.8039	0.8242	0.7888	0.8364	0.8439	0.8290	0.7027
Stacking-FFNN	0.8121	0.7939	0.8330	0.8421	0.8162	0.8696	0.7139

Among the individual transformer-based models, RoBERTa achieved the highest F1-macro score of 0.7968, outperforming strong baselines such as DeBERTa-v3 with F1-macro score of 0.7895. Notably, this score also exceeds the reference values reported in the original dataset paper by Rahman et al. (2024), where the benchmarks were 0.76 F1-macro and 0.80 F1-micro.

Beyond single models, ensemble learning approaches demonstrated clear performance gains. In particular, the Stacking-FFNN method attained the best results, with F1-macro of 0.8121 and F1-micro of 0.8421, surpassing both individual transformer models and simpler ensemble strategies such as hard and soft voting. Ensemble learning methods are used as a powerful solution to prevent overfitting by leveraging the complementary strengths of different models and thus reducing model-specific bias and variance. In ensemble learning models, the selection of base classifiers and their ability to accommodate diversity in their decisions are as important as how these models are combined. Hard Voting explicitly combines the decision classes of individual models, providing more robust predictions, thereby smoothing out model-specific errors. However, stacking, another more intelligent model fusion strategy, can capture nonlinear relationships between base model outputs and yield higher performance on challenging tasks such as depression classification. Unlike voting-based methods, stacking learns how base model predictions relate to actual outputs and captures nonlinear dependencies between probability distributions. Furthermore, when stacking is combined with an FFNN, it results in a more robust model that learns how to optimally weight and integrate the predictions of the base models. Consequently, Stacking-FFNN simultaneously corrects for systematic biases inherent in individual models while reducing model variance that contributes to overfitting. These advantages are particularly valuable for multi-label depression detection, where textual inputs often contain overlapping emotional cues, high inter-label correlation, and significant noise. Consequently, the Stacking-FFNN ensemble achieved superior performance because it is better suited to model the noisy nature of the DepressionEmo dataset.

Overall, the findings highlight that ensemble methods built upon transformer-based encoders consistently outperform individual models in challenging NLP tasks like multi-label emotion classification. Notably, combining diverse transformer outputs with a neural network meta-learner provides a significant performance boost. From a practical perspective, this is especially important in psychology, where recognizing and analyzing multiple co-occurring emotional states -rather than a single dominant emotion- is critical for more accurate and meaningful assessments.

To provide a more comprehensive evaluation of the experimental results, Table 4 includes the emotion predictions produced by the proposed model for selected textual data examples, in addition to the standard classification performance metrics, allowing for a qualitative discussion of the experimental outcomes.

**Table 5:** Qualitative analysis of model predictions showing instances of exact match, partial error, and failure cases

Example	ID	Post Content	True labels	Predicted labels	Jaccard acc
1	e38ntt	Loneliness is terrible ### It feels like nobody wants me. All my friends live hours away and I rarely talk to them. If we do talk, it’s very superficial and fake feeling. I just want someone I can share things with, other than arbitrary, superficial, nonsense. Idk why I’m writing this. I’m just so lonely. It hurts so much! I don’t want people to feel bad for me, I just want someone to listen to me and care for me. I guess this is more of a journal entry than anything else.	emptiness, hopelessness, loneliness, sadness, worthlessness	emptiness, hopelessness, loneliness, sadness, worthlessness	1.00
2	ryjtfw	Psychiatrist wrote in my notes that I was unkempt ### Hi everyone. I’m a 23 year old female. I struggle with mental health issues. I have OCD. I had an appointment with a new psychiatrist yesterday. I really liked her, much better than the nurse practitioner I had during the previous appointment. I found out that I have major depressive disorder. I also found out that I most likely have PTSD, which is something no one had ever told me before. When I was looking at my after visit notes today, I saw that the psychiatrist had written that I was unkempt. It kind of hurt my feelings. I mean I know I’ve been looking like a ### ever since I’ve been depressed, but why rub it in? I already know I look bad. I kind of wished I hadn’t even looked at my notes because of seeing that comment. Has anyone else experienced this?	anger, hopelessness, sadness, worthlessness	hopelessness, sadness, worthlessness	0.75
3	179fkof	Is it me or is it depression that stuns my growth for success? ### I was never bright in school. I barely graduated from high school. I had a few jobs afterwards, but no career. I am almost 40 years old. I have no wife and no kids. I never thought I had depression. The moment I started typing this paragraph, I starting to get depressed.	sadness	emptiness, hopelessness, sadness, worthlessness	0.25
4	17csiq0	Medication ### I have tried so many medication‘s for my anxiety and depression. They all give me the same effect I can’t laugh. I can’t cry. I can’t smile. I don’t feel sad but I don’t feel joy either. I’m zoned out, and I feel like a zombie. I don’t even feel human, and it not to mention it makes me so exhausted and sleepy. I cannot focus or function on any meds I’ve tried so far. Has anyone else gone through this and finally found something that seemed to help?	hopelessness	cognitive dysfunction, emptiness, hopelessness, loneliness, sadness, worthlessness	0.17

Table 5 presents representative test samples illustrating the proposed model’s prediction behavior. In the first example (ID: e38ntt), multiple negative emotions are expressed directly, and the model correctly predicts all ground-truth labels. It achieves a Jaccard score of 1.00. This indicates that the model can detect emotions that appear together when explicitly expressed. For the second post (ID: ryjtfw), the model correctly predicted most of the true labels but missed the anger label. As a result, the Jaccard score was calculated as 0.75. While feelings of hopelessness, sadness, and worthlessness are conveyed more directly in the text, anger is expressed more indirectly. This example shows that while some emotions are expressed more explicitly in the text, implicit emotions are more likely to be missed by the model. In the third example (ID: 179fkof), although the ground-truth label was only sadness, the model predicted emptiness, hopelessness, and worthlessness in addition to sadness; therefore, the Jaccard score was 0.25. Because the text conveyed a general negativity about life and the future rather than directly stating a single emotion, the model predicted semantically similar labels in addition to sadness. This suggests that the model may overpredict in texts where emotional cues are presented within a broader context. Finally, in the fourth example (ID: 17csiq0), although the ground-truth label was only hopelessness, the model also predicted cognitive dysfunction, emptiness, loneliness, sadness, and worthlessness, resulting in a low Jaccard score of 0.17. Similar to the third example, this suggests that broad and overlapping cues in the text can lead the model to assign multiple related emotions rather than a single specific label. In the fourth post, expressions like difficulty focusing, reduced functioning, and emotional numbness can fit several labels. For this reason, the model predicts multiple related emotions instead of a single label. Overall, our proposed model produces more consistent results in texts where emotions are expressed explicitly and strongly, but it may assign additional emotion labels when cues are implicit.

### Comparison with literature

In this section, the findings from our study are compared with the results reported in the existing literature that used the DepressionEmo dataset, and the similarities and differences are discussed in Table 6.

**Table 6:** Comparison with existing literature

Reference	Method	Performance (F1 Score)
(Rahman et al. 2024)	SVM Light GBM XGBoost GAN-BERT BERT-cased BERT-uncased BART T5-small T5-base	0.61 (Micro) 0.65 (Micro) 0.66 (Micro) 0.75 (Micro) 0.79 (Micro) 0.80 (Micro) 0.80 (Micro) 0.75 (Micro) 0.79 (Micro)
(Khan et al. 2024)	Contrastive Learning+RoBERTa	0.7816 (Average)
(Violides et al. 2024)	Fine-tuned RoBERTa	0.81 (Average)
(Younas et al. 2025)	Stacked LSTM	0.70 (Average)
Our study	Stacking-FFNN	0.8421 (Micro) 0.8121 (Macro)

Studies using the DepressionEmo dataset show that F1 Scores reported in the literature generally range from 0.61 to 0.81, depending on the method used. Rahman et al. (2024) compared classical ML methods with various transformer-based models and reported that Micro F1 Scores ranged from 0.61 to 0.80, with the highest performance achieved by the BERT-uncased and BART models. Khan et al. (2024) achieved an average F1 Score of 78.16% with the contrastive learning + RoBERTa approach, while Violides et al. (2024) achieved an average F1 Score of 81.00% with the fine-tuned RoBERTa model. Furthermore, Younas et al. (2025) reported an F1 Score of 0.70 using the Stacked LSTM model.

Compared to these studies, the proposed Stacking-FFNN approach, with a Macro F1 value of 0.8121, appears to outperform or even compete with many transformer-based singleton models in the literature. This result demonstrates that the model offers balanced inter-class performance and strong generalization ability. Furthermore, the Micro F1 Score of 0.8421 demonstrates the model’s high overall classification accuracy and that the stacking structure effectively integrates complementary information obtained from different models. Although direct comparison is challenging due to differences in evaluation metrics, the results suggest that the proposed approach is competitive with, and in some cases exceeds, the performance of existing state-of-the-art models reported in the literature. While comparing the proposed method with existing methods in the literature is difficult due to differences in evaluation criteria, it is clearly seen that the proposed method is competitive with, and in some cases even exceeds, the performance of the state-of-the-art models currently available.

### Limitations

While BERT-based architectures demonstrate high performance in learning semantic representations, their black-box nature makes it difficult to clearly and interpretably explain the underlying reasons for their decisions. This presents a significant limitation, particularly in clinically critical applications. The performance and generalization capabilities of BERT and its derivative models depend heavily on the quality and scope of the data they are trained on. If the dataset does not adequately reflect the diversity of depressive symptoms and emotional expressions across different demographic and sociocultural groups, the generalization success of the developed classification models can be negatively impacted. While domain-specific models such as MentalBERT and MentalRoBERTa are fine-tuned for specific psychological tasks, they can sometimes fall short in capturing implicit meanings and subtle linguistic nuances (Garg et al. 2024). While evaluating model performance on unseen data is critical, such evaluations can also lead to results that do not fully reflect real-world conditions.

Another important limitation concerns the DepressionEmo dataset used in this study. While the DepressionEmo dataset used in this study has been used for academic purposes on the automatic classification of depression, it was not derived from a real patient-physician interview. The dataset consists of user generated comments, which limits its clinical validity. The depression emotion labels, and especially suicide intent in the dataset are based on user statements and annotator interpretations rather than formal clinical diagnoses. This carries the risk of label noise and subjective interpretation. Therefore, models trained with DepressionEmo are limited in their ability to perform diagnoses on real clinical data.

Additionally, transformer-based language models used in this study were trained on Reddit posts that carry some significant risks. Texts produced on online platforms like Reddit can contain everyday language, exaggeration, irony, humor, and community-specific discourse patterns. This can lead to the models becoming overly accommodating to platform-specific language use and failing to accurately generalize mental states expressed in clinical or real-world contexts. Particularly with sensitive labels such as suicidal intent, this type of bias increases the risk of misinterpretation and requires ethical caution.

Furthermore, the lack of universal representation of demographic variables such as age, gender, and cultural background in the data source used is one of the main limitations of the study (Huang et al. 2023). Platform-specific language use, irony, and lack of context can lead to misinterpretations of the texts (Das et al. 2024).

Finally, false positive or false negative predictions, particularly those related to suicide intent, pose significant ethical risks. False positive predictions can lead to unnecessary labeling and stigmatization of individuals, while false negative predictions can cause real risks to be overlooked. Therefore, the proposed model should be considered solely as a decision support tool, not for clinical or automated decision-making mechanisms.

## Conclusion

In this study, we addressed the complex task of detecting co-occurring emotions related to depression in social media texts by leveraging transformer-based encoders and ensemble learning techniques. Using the DepressionEmo dataset, we systematically compared the performance of several pre-trained models, including RoBERTa, Mental-BERT, Mental-RoBERTa, DistilBERT, and DeBERTa-v3. While individual transformer models demonstrated strong results, our experiments confirmed that ensemble strategies significantly enhance performance. In particular, the proposed stacking approach with a FFNN achieved the highest scores across evaluation metrics, surpassing both individual models and simpler ensemble techniques such as hard and soft voting.

The findings highlight the practical value of ensemble learning in improving the robustness and reliability of depression-related emotion detection. By combining diverse model outputs, the ensemble framework effectively captures subtle and interdependent emotional patterns, which is essential for more accurate identification of complex psychological states. In this respect, the study provides a methodological foundation for computational research in mental health.

However, it should be specifically emphasized that this study is not diagnostic and the results obtained should not be interpreted as a substitute for clinical decisions. The study was conducted using a public and anonymized social media dataset, and the proposed approach only provides a framework for research and decision-support purposes. Direct use in clinical settings will only be possible after prospective clinical trials, ethics committee approvals, data governance policies, and relevant regulatory requirements are met.

Future studies could focus on examining the clinical validity of the method across different populations and data sources, integrating explainability methods, and expanding the model with multimodal data. Such studies would contribute to the safe and responsible evaluation of the approach in real-world mental health research.

## Data Availability

The dataset used in this study is public.

## References

[CR1] Abbas MA, Munir K, Raza A, Samee NA, Jamjoom MM, Ullah Z (2024) Novel transformer based contextualized embedding and probabilistic features for depression detection from social media. IEEE Access

[CR2] Adarsh V, Kumar PA, Lavanya V, Gangadharan GR (2023) Fair and explainable depression detection in social media. Inf Process Manag 60(1):103168

[CR3] Angskun J, Tipprasert S, Angskun T (2022) Big data analytics on social networks for real-time depression detection. J Big Data 9(1):69 35610999 10.1186/s40537-022-00622-2 PMC9121859

[CR4] Arriba-Pérez F, García-Méndez S (2024) Detecting anxiety and depression in dialogues: a multi-label and explainable approach. arXiv preprint arXiv:2412.17651

[CR5] Ashiga M, Jie W, Wu F, Voskanyan V, Dinmohammadi F, Brookes P, Wang Z (2025) Ensemble learning for large language models in text and code generation: a survey. arXiv preprint arXiv:2503.13505

[CR6] Beniwal R, Saraswat P (2024) A hybrid BERT-CPSO model for multi-class depression detection using pure hindi and hinglish multimodal data on social media. Comput Electr Eng 120:109786

[CR7] Cha J, Kim S, Park E (2022) A lexicon-based approach to examine depression detection in social media: the case of Twitter and university community. Humanit Soc Sci Commun 9(1):1–10 10.1057/s41599-022-01313-2 PMC9491270 36159708

[CR8] Chiong R, Budhi GS, Dhakal S, Chiong F (2021) A textual-based featuring approach for depression detection using machine learning classifiers and social media texts. Comput Biol Med 135:104499 34174760 10.1016/j.compbiomed.2021.104499

[CR9] Das AK, Mishra D (2024) The automation of sentiment analysis on cross-platform social media data: a comparative study of techniques and tools. Library of Progress-Library Science, Information Technology & Computer, p 44

[CR10] Dennstädt F, Zink J, Putora PM, Hastings J, Cihoric N (2024) Title and abstract screening for literature reviews using large language models: an exploratory study in the biomedical domain. Syst Rev 13(1):158 38879534 10.1186/s13643-024-02575-4 PMC11180407

[CR11] DistilBERT (2025) Distilbert-base-uncased [Model]. Hugging Face. https://huggingface.co/distilbert/distilbert-base-uncased

[CR12] Facebook AI (2025) RoBERTa-base [Model]. Hugging Face. https://huggingface.co/FacebookAI/roberta-base

[CR13] Fang C, Li X, Fan Z, Xu J, Nag K, Korpeoglu E, Achan K (2024a) Llm-ensemble: optimal large language model ensemble method for e-commerce product attribute value extraction. In Proceedings of the 47th international ACM SIGIR conference on research and development in information retrieval. pp 2910–2914

[CR14] Fang X, Xu W, Tan FA, Zhang J, Hu Z, Qi Y, Faloutsos C (2024b) Large language models (LLMs) on tabular data: prediction, generation, and understanding–a survey. arXiv preprint arXiv:2402.17944

[CR15] Farruque N, Huang C, Zaiane O, Goebel R (2019), April Basic and depression specific emotions identification in tweets: multi-label classification experiments. In: International conference on computational linguistics and intelligent text processing. Springer, Cham, pp 293–306

[CR16] Garg M, Sathvik MSVPJ, Raza S, Chadha A, Sohn S (2024) Reliability analysis of psychological concept extraction and classification in user-penned text. In: Proceedings of the international AAAI conference on web and social media. Vol. 18, pp 422–434 10.1609/icwsm.v18i1.31324 PMC11881108 40046688

[CR17] Ghosh S, Ekbal A, Bhattacharyya P (2022) A multitask framework to detect depression, sentiment and multi-label emotion from suicide notes. Cogn Comput 14(1):110–129 10.1038/s41598-022-08438-z PMC8923342 35292695

[CR19] Haque R, Khan MA, Rahman H, Khan S, Siddiqui MIH, Limon ZH, Swapno SMMR, Appaji A (2025) Explainable deep stacking ensemble model for accurate and transparent brain tumor diagnosis. Comput Biol Med 191:110166 40249992 10.1016/j.compbiomed.2025.110166

[CR20] Hassan AA, Hanafy RJ, Fouda ME (2024) Automated multi-label annotation for mental health illnesses using large language models. arXiv preprint arXiv:2412.03796

[CR18] Hämäläinen M, Patpong P, Alnajjar K, Partanen N, Rueter J (2021) Detecting depression in Thai blog posts: a dataset and a baseline. arXiv preprint arXiv:2111.04574

[CR21] He P, Liu X, Gao J, Chen W (2020) Deberta: decoding-enhanced bert with disentangled attention. arXiv preprint arXiv:2006.03654

[CR22] Hinojosa Lee MC, Braet J, Springael J (2024) Performance metrics for multilabel emotion classification: comparing micro, macro, and weighted f1-scores. Appl Sci 14(21):9863

[CR23] Holtzman A, West P, Shwartz V, Choi Y, Zettlemoyer L (2021) Surface form competition: why the highest probability answer isn’t always right. arXiv preprint arXiv:2104.08315.

[CR24] Huang C, Bandyopadhyay A, Fan W, Miller A, Gilbertson-White S (2023) Mental toll on working women during the COVID-19 pandemic: An exploratory study using Reddit data. PloS one 18(1):e0280049 36649225 10.1371/journal.pone.0280049 PMC9844921

[CR25] Hugging Face (2019) Pretrained models — Transformers 2.4.0 documentation. https://huggingface.co/transformers/v2.4.0/pretrained_models.html

[CR26] Hugging Face (2022) microsoft/deberta-v3-base model. https://huggingface.co/microsoft/deberta-v3-base

[CR27] Hugging Face (2024) distilbert-base-multilingual-cased model. https://huggingface.co/distilbert/distilbert-base-multilingual-cased

[CR28] Ji S, Zhang T, Ansari L, Fu J, Tiwari P, Cambria E (2021) Mentalbert: publicly available pretrained language models for mental healthcare. arXiv preprint arXiv :211015621

[CR29] Khan PI, Dengel A, Ahmed S (2024) Improving disease detection from social media text via self-augmentation and contrastive learning. arXiv preprint arXiv:2405.01597

[CR30] Kim J, Park O, Park E (2025) MOSS-6: a multi-label dataset and deep learning model for detecting diverse social support-seeking behaviours in online mental health communities. Information, Communication & Society. 10.1080/1369118x.2025.2460558

[CR31] Kumar M, Nigam A, Singh SV (2024) Analysis of hybrid fuzzy-neuro model for diagnosis of depression. In: Recent advances in computational intelligence and cyber security. CRC, pp 36–42

[CR32] Lam L, Suen CY (1994) A theoretical analysis of the application of majority voting to pattern recognition. In: Proceedings of the 12th IAPR international conference on pattern recognition, Vol. 3-conference C: signal processing (Cat. No. 94CH3440-5). IEEE, Vol. 2, pp 418–420

[CR33] Liu Y (2024) Depression detection via a Chinese social media platform: a novel causal relation-aware deep learning approach. J Supercomput 80(8):10327–10356

[CR34] Liu Y, Ott M, Goyal N, Du J, Joshi M, Chen D, Stoyanov V (2019) Roberta: a robustly optimized bert pretraining approach. arXiv preprint arXiv:1907.11692

[CR35] Liu J, Chen W, Wang L, Ding F (2024) A hybrid depression detection model and correlation analysis for social media based on attention mechanism. Int J Mach Learn Cybern 15(7):2631–2642

[CR36] Lu J, Pang Z, Xiao M, Zhu Y, Xia R, Zhang J (2024) Merge, ensemble, and cooperate! A survey on collaborative strategies in the era of large language models. arXiv preprint arXiv :240706089

[CR37] Mental (2025a) mental-roberta-base [Model]. Hugging Face. https://huggingface.co/mental/mental-roberta-base

[CR38] Mental (2025b) mental-bert-base-uncased [Model]. Hugging Face. https://huggingface.co/mental/mental-bert-base-uncased

[CR39] Microsoft (2025) DeBERTa-v3-base [Model]. Hugging Face. https://huggingface.co/microsoft/deberta-v3-base

[CR40] Neagu AC, Spinu S (2024) Detection of mental health condition using machine learning algorithms. J Milit Technol. 10.32754/jmt.2024.2.04

[CR41] Palmon N, Momen S, Leavy M, Curhan G, Boussios C, Gliklich R (2021) PMH52 use of a natural language processing-based approach to extract suicide ideation and behavior from clinical notes to support depression research. Value Health 24:S137

[CR42] Pande SD, Hasane Ahammad SK, Gurav MN, Faragallah OS, Eid MM, Rashed ANZ (2024) Depression detection based on social networking sites using data mining. Multimedia Tools Appl 83(9):25951–25967

[CR44] Park D, Lee G, Kim S, Seo T, Oh H, Kim SJ (2024) Probability-based multi-label classification considering correlation between labels–focusing on DSM-5 depressive disorder diagnostic criteria. IEEE Access. 10.1109/access.2024.340170439726803

[CR43] Park D, Lim S, Choi Y, Oh H (2023) Depression emotion multi-label classification using everytime platform with DSM-5 diagnostic criteria. IEEE Access 11:89093–89106

[CR45] Phiri D, Makowa F, Amelia VL, Phiri YVA, Dlamini LP, Chung MH (2025) Text-based depression prediction on social media using machine learning: systematic review and meta-analysis. J Med Internet Res 27:e59002 40215481 10.2196/59002 PMC12032503

[CR46] Polikar R (2006) Ensemble based systems in decision making. IEEE Circuits Syst Mag 6(3):21–45

[CR47] Pradnyana GA, Anggraeni W, Yuniarno EM, Purnomo MH (2025) Revealing Depression Through Social Media via Adaptive Gated Cross-Modal Fusion Augmented With Insights From Personality Traits. IEEE Access. 10.1109/access.2025.3593273

[CR48] Rahman ABS, Ta HT, Najjar L, Azadmanesh A, Gönul AS (2024) Depressionemo: a novel dataset for multilabel classification of depression emotions. J Affect Disord 366:445–458 39214375 10.1016/j.jad.2024.08.013

[CR49] Ren X, Burkhardt HA, Areán PA, Hull TD, Cohen T (2024) Deep representations of first-person pronouns for prediction of depression symptom severity. In: AMIA annual symposium proceedings. Vol. 2023, p 1226 PMC10785936 38222407

[CR50] Sanh V, Debut L, Chaumond J, Wolf T (2019) DistilBERT, a distilled version of BERT: smaller, faster, cheaper and lighter. arXiv preprint arXiv:1910.01108

[CR51] Shah SM, Gillani SA, Baig MSA, Saleem MA, Siddiqui MH (2025) Advancing depression detection on social media platforms through fine-tuned large language models. Online Soc Netw Media 46:100311

[CR52] Siddiqui MIH, Khan S, Limon ZH, Rahman H, Khan MA, Al Sakib A, Swapno SMMR, Haque R, Reza AW, Appaji A (2025) Accelerated and accurate cervical cancer diagnosis using a novel stacking ensemble method with explainable AI. Informatics in Medicine Unlocked 56:101657

[CR53] Thekkekara JP, Yongchareon S, Liesaputra V (2024) An attention-based CNN-BiLSTM model for depression detection on social media text. Expert Syst Appl 249:123834

[CR54] Tong L, Liu Z, Jiang Z, Zhou F, Chen L, Lyu J, Zhou H (2022) Cost-sensitive Boosting Pruning Trees for depression detection on Twitter. IEEE Trans Affect Comput 14(3):1898–1911

[CR55] Vance LA, Way L, Kulkarni D, Palmer EO, Ghosh A, Unruh M, Sarkar J (2025) Natural language processing to identify suicidal ideation and anhedonia in major depressive disorder. BMC Med Inf Decis Mak 25(1):20 10.1186/s12911-025-02851-w PMC11730826 39806393

[CR56] Violides M, Timtong T, Bezdek K, Andreadis P (2024) Impact of COVID-19 on linguistic expression of depression in online communities. In: Proceedings of the 2024 5th international symposium on artificial intelligence for medicine science. pp 787–793

[CR57] Willis R, Du Y, Leibo JZ, Luck M (2025) Will systems of llm agents cooperate: an investigation into a social dilemma. arXiv preprint arXiv :250116173

[CR58] Wolpert DH (1992) Stacked generalization. Neural Netw 5(2):241–259

[CR59] World Health Organization (2023) Depressive Disorder (depression). https://www.who.int/news-room/fact-sheets/detail/depression

[CR60] Wu JL, He Y, Yu LC, Lai KR (2020) Identifying emotion labels from psychiatric social texts using a bi-directional LSTM-CNN model. IEEE Access 8:66638–66646

[CR61] Yang C, Wang Z, Tan W, Tan Z, Ji C, Zhou Z (2025) Hierarchical dual-head model for suicide risk assessment via MentalRoBERTa. arXiv preprint arXiv:2510.20085

[CR62] Yoo DW, Shi JM, Rodriguez VJ, Saha K (2025) AI chatbots for mental health: values and harms from lived experiences of depression. arXiv preprint arXiv:2504.18932

[CR63] Younas A, Riaz S, Ali S, Khan R, Ullah M, Kwak D (2025) Stacked LSTM Model for Contextual Correlation Detection Among Multiple Emotions. IEEE Access. 10.1109/access.2025.3582764

[CR64] Zandbiglari K, Kumar S, Bilal M, Goodin A, Rouhizadeh M (2025) Enhancing suicidal behavior detection in EHRs: A multi-label NLP framework with transformer models and semantic retrieval-based annotation. J Biomed Inform 161:104755 39631489 10.1016/j.jbi.2024.104755

